# The role of explanatory models of breast cancer in breast cancer prevention behaviors among Arab-Israeli physicians and laywomen

**DOI:** 10.1017/S1463423620000237

**Published:** 2020-11-03

**Authors:** Michal Soffer, Miri Cohen, Faisal Azaiza

**Affiliations:** School of Social Work, Faculty of Social Welfare & Health Sciences, University of Haifa, Haifa 3498838, Israel

**Keywords:** adherence, cancer-related perceptions, explanatory models, mammography, physicians’ recommendations, screening

## Abstract

**Background:**

‘Explanatory Models’ (EMs) are frameworks through which individuals and groups understand diseases, are influenced by cultural and religious perceptions of health and illness, and influence both physicians and patients’ behaviors.

**Aims:**

To examine the role of EMs of illness (cancer-related perceptions) in physicians’ and laywomen’s behaviors (decision to recommend undergoing regular mammography, adhering to mammography) in the context of a traditional-religious society, that is, the Arab society in Israel.

**Methods:**

Two combined samples were drawn: a representative sample of 146 Arab physicians who serve the Arab population and a sample composed of 290 Arab women, aged 50–70 years, representative of the main Arab groups residing in the north and center of Israel (Muslims, Christians) were each randomly sampled (cluster sampling). All respondents completed a closed-ended questionnaire.

**Results:**

Women held more cultural cancer-related beliefs and fatalistic beliefs than physicians. Physicians attributed more access barriers to screening as well as fear of radiation to women patients and lower social barriers to screening, compared with the women’s community sample. Higher fatalistic beliefs among women hindered the probability of adherence to mammography; physicians with higher fatalistic beliefs were less likely to recommend mammography.

**Conclusions:**

The role of cultural perceptions needs to be particularly emphasized. In addition to understanding the patients’ perceptions of illness, physicians must also reflect on the social, cultural, and psychological factors that shape their decision to recommend undergoing regular mammography.

## Introduction

Breast cancer is the second leading cause of death from cancer among women in Western countries (Ferlay *et al*., 2010). Breast cancer survival, to a large extent, depends on its early detection. Despite recent questioning about regular mammography screening – due to high over-diagnosis and false-positive rates in Western countries (Gøtzsche and Jørgensen, 2013), systematic screening programs, especially for average-risk women aged 50 years and over, have been shown to significantly increase early detection and reduce mortality (Oeffinger *et al*., 2015). Average-risk women, aged 50–74 years and above, are currently being advised to engage in early detection practices by undergoing regular mammography testing once every two years (Israel Cancer Association, 2015).

The Arab population in Israel is an ethnic minority, constituting about 20% of the Israeli population. This population includes several religious groups: 83% Muslims, 8% Christians, 8% Druze, and 1% other (Central Bureau of Statistics, 2015). In spite of the diversity of religions, the Arab people share common history, culture, and tradition and express similarity in their way of life (Zoabi and Savaya, 2017). Although the Arab population is currently experiencing modernization processes, it is still, to a large extent, a traditional and religious society (Cohen, 2014). Breast cancer incidence has been found to be significantly lower among Arab women (Christians and Muslims) compared to Jewish women in Israel and women in Western countries, but Arab women are more frequently diagnosed at a more advanced stage of the disease (Cohen, 2014). It has been suggested that this tendency toward a later diagnosis could be attributed to the significantly lower rate of regular screening for the early detection of breast cancer among Arab women (Keinan-Boker *et al*., 2013) and is predominantly related to social and cultural screening barriers (Azaiza and Cohen, 2008; Cohen and Azaiza, 2008; Azaiza *et al*., 2010; 2011). Largely due to the provision and promotion of the free-of-charge screenings and intensive efforts of the Israeli health services and the Israel Cancer Society, the majority of Arab women aged 50 years and over have undergone a mammography screening at least once. This has had an impact on narrowing the screening rate gap between Arab and Jewish women (Keinan-Boker *et al*., 2013). However, the general rate of adherence to the recommended mammography schedule (undergoing mammography every two years) is still low among Arab women (Azaiza *et al*., 2011).

Adherence to screening for the early detection of breast cancer was found to be predicted by health beliefs, especially perceived benefits of screening and perceived barriers to screening (Champion, 1999), such as fear of pain or of the damage caused by radiation (Cohen and Azaiza, 2008). Several studies among Arab Muslim and Christian individuals elicited culture bounded perceptions of cancer and barriers to screening, including traditional beliefs concerning cancer, barriers related to exposure of the body, access barriers, and cancer fatalism (Cohen and Azaiza, 2008; Cohen, 2013). These cultural perceptions predicted adherence to mammography, even after controlling for various demographic variables (Azaiza *et al*., 2011). However, in a comparison between women from the Palestinian Authority (a traditional society) and Arab women in Israel (a traditional society that has adopted a more Western lifestyle), both groups consisting of Muslims and Christians, the cultural beliefs were substantially higher among the Palestinian Authority participants (Azaiza *et al*., 2010; 2011).

Anthropologist and psychiatrist Arthur Kleinman (1980) coined lay or patients’ understanding or perceptions of illness ‘Explanatory Models’ (EMs). According to Kleinman (1980), EMs are frameworks through which individuals and groups understand diseases. A patient’s EM of an illness, according to Kleinman *et al*. (2006), consists of his/her beliefs and perceptions of the illness, the individual and social meaning that the illness holds for him/her, his/her expectations concerning what will happen to him/her as well as the physician’s actions, and his/her own therapeutic goals. They further argued that physicians’ EMs may differ from those of their patients, while such discrepancies – if not addressed by accounting for the patient’s model and communicating the physician’s EM in a way that allows the patient to understand and accept the EM – might interfere with clinical care.

Kleinman *et al*. (2006) argued that:

[c]ontemporary medical practice has become increasingly discordant with lay expectations. Modern physicians diagnose and treat diseases (abnormalities in the structure and function of body organs and systems), whereas patients suffer illnesses (experiences of disvalued changes in states of being and in social function; the human experience of sickness). (Kleinman *et al*., 2006:140–141)

Based on Kleinman’s seminal work, Ashton and colleagues’ (2003) ‘integrative model’ of patient–physician communication in the case of cultural discord between physician and patient suggests that the EM of an illness formulates the behaviors of both the patient (i.e., illness behavior) and the physician. They further argue that discordance between the physician’s and patient’s EMs impacts the patient’s behavior and, consequently, his/her health outcomes. It is worth noting that this assumption was supported by Rich and colleagues’ (2002) qualitative study of EM, which focused on asthma and health-related behaviors among children and youth with asthma.

Comparative studies of EM of illnesses between physicians and patients have yielded mixed results. Ample research supports the fact that physicians have different EMs or perceptions of illness than their patients (e.g., Haidet *et al*., 2008; Weller *et al*., 2012; Nowicka-Sauer *et al*., 2016), even if they share the same language and culture (see, e.g., Aido and Harpham, 2001; Weller *et al*., 2012). These studies’ findings imply that medicine is a type of ‘culture’ as it is characterized by the biomedical understanding of the body and pathology (Kleinman, 1980; Schouten and Meeuwesen, 2006; Tirodkar *et al*., 2011; Lupton, 2012), whereas patients’ EMs are influenced by, among other things, culture (Kleinman *et al*., 2006).

Nevertheless, there are studies that did not find differences between laypersons or community samples and physicians’ EMs. A study, for example, which explored EMs of AIDS among US and Mexican communities, as well as among physicians from both of these countries, found a core and similar biomedical EM of AIDS in all four subsamples (Baer *et al*., 2004). Similarly, a study which examined the EM of the common cold between physicians and laypersons in Texas and Mexico found that they all shared a common EM for this disease (Baer *et al*., 2008).

In this study, we examined the role of EMs of illness (cancer-related perceptions) in physicians’ and laywomen’s behaviors.

Specifically, our aims were

1. To compare cancer-related perceptions and perceptions of women’s barriers to screening between Arab physicians and Arab laywomen.
2. To examine the role of cancer-related perceptions in Arab physicians’ decisions to recommend undergoing regular mammography screening and in Arab women’s reports of adhering to mammography screening.

## Methods

### Participants

This study is a secondary analysis of two surveys. Both surveys received the approval of the Ethics board of the Faculty of Health and Social Welfare Sciences at the University of Haifa (approval #232/13, for the physician’s survey; approval #128/13, for the women survey). In this study, the two independent samples from the aforementioned surveys were combined and compared as follows.

#### Physicians’ sample

The sample was composed of 146 Arab physicians who were employed in community health services (family physicians, gynecologists, or internal medicine physicians), recruited from 14 communities in the northern, central, and southern regions of Israel, including Arab cities and villages and mixed Jewish-Arab cities, sampled by the multi-stage sampling method. According to the size of each community, 5–20 participants were randomly recruited from each. The sampling frame was the list of physicians registered in the Israeli Medical Association. We telephoned the randomly sampled physicians, explained the study aims and procedure, and scheduled a close-ended interview. Prior to the interview, participants signed an informed consent form. Non-answered calls were attempted several times. Out of the 320 physicians who were approached and asked to participate in a survey on early detection of breast cancer among their patients, 146 agreed to participate (a 47% participation rate).

#### Women’s community sample

A representative sample of the main Arab groups residing in northern, central, and southern Israel (Muslims, Christians) was drawn. The sample composed of 290 Arab women between the age 50 and 70 years. First, a random sample of ten cities and villages in northern, central, and southern Israel was conducted. Second, a random sample of women was drawn from Arab phone directories within each sampled city or village. The number of women that were sampled was contingent on the size of the population within the sampled city or village. Eligibility criteria were as follows: age 50–70 years, not being ill with cancer in the past or present, and fluent in Arabic. We phoned the women and invited them to participate in a survey on early detection of breast cancer and scheduled a close-ended interview. Prior to the interview, the women signed an informed consent form. Non-answered calls were attempted several times. The response rate was 90.9%.

### Questionnaires

#### Women’s questionnaire

The women’s questionnaire composed of questions pertaining to socio-demographic characteristics; adherence to screening guidelines; cultural beliefs regarding cancer, social and access barriers to screening, fear of pain; fear of radiation; and fatalistic beliefs.

*Socio-Demographic characteristics included* age, family status, education, perceived economic status, and perceived level of religiosity.

*Adherence to screening guidelines* included questions on mammography frequency, and the last time the participant underwent mammography. Adherence to guidelines was defined as being on time with mammography, namely, having undergone a mammography examination every two years, with the last mammogram having been conducted not later than two years ago for women aged 52+ years, and having undergone at least one mammogram if the woman was between 50 and 52 years of age.

#### Cultural beliefs regarding cancer, social, and access barriers to screening

Cultural beliefs and the two types of barriers to screening (social and access) are three sub-scales of the Arab culture-specific barriers (ACSB) questionnaire (Appendix 1) (Cohen and Azaiza, 2008) that was previously validated and found with good validity and reliability indices (for details concerning development, reliability, and validity, see Cohen and Azaiza, 2008). The questionnaire was used in several studies (e.g., Azaiza *et al*., 2010; 2011).

*Women’s cultural beliefs regarding cancer*: This subscale of the ACSB comprises of items related to cultural beliefs regarding breast cancer (e.g., prayers help the healing process, traditional medicine helps, cancer is a punishment for one’s personal sins, cancer is one of God’s tests). Participants were asked to rate each question on a scale from 1 = not at all to 5 = very much. The internal consistency of this subscale was .91 (Cronbach’s alpha).

*Women’s social barriers to screening*: This subscale of the ACSB comprises of items probing perceptions of stigma and shame related to breast cancer (e.g., fear pity of others, fear of losing friends). Participants were asked to rate each question on a scale from 1=not at all to 5=very much. The internal consistency of this subscale was 0.93 (Cronbach’s alpha).

*Women’s access barriers to screening*: This subscale of the ACSB comprises of questions pertaining to environmental barriers (distance, communication barriers, financial expenses), exposure barriers (being examined by a male/female physician, religious barriers related to not wanting to expose the body (modesty issues), the fear of being seen in a breast clinic). Participants were asked to rate each question on a scale from 1=not at all to 5=very much. The internal consistency (Cronbach’s alpha) was .84.

*Fear of pain* was assessed by an item probing the degree of fear of the pain experienced during mammography. Participants were asked to rate each question on a scale from 1 = not at all to 5 = very much.

*Fear of radiation* was assessed by an item probing the degree of fear from the radiation caused by mammography. Participants were asked to rate each question on a scale from 1 = not at all to 5 = very much.

*The fatalistic beliefs scale* (Azaiza *et al*., 2010; 2011) was composed of four items: two items that related to fatalistic beliefs about breast cancer (e.g., breast cancer is a death sentence, despite the treatment); two items related to avoidance of cancer (i.e., it is better not to do the screening; ‘what you don’t know can’t hurt you’). Participants were asked to rate each question on a scale from 1 = not at all to 5 = very much. Internal consistency was .78 (Cronbach’s alpha).

#### Physicians’ questionnaires

The physician’s questionnaire composed of questions pertaining to socio-demographic characteristics, patterns of recommending and discussing screening facilitators and barriers with patients, physicians’ cultural beliefs regarding cancer, perceived patients’ social and access barriers to screening, perceived patients’ fear of mammography-related pain and radiation, and fatalistic beliefs.

*Socio-demographic characteristics* included gender, age, family status, education, years of seniority as a physician, and specialty.

*Patterns of recommending and discussing screening facilitators and barriers with patients* consisted of five separate items examining the rate of recommending biannual mammography to women aged 50–70 years and discussing the barriers to screening and screening advantages with women who refused to attend the exams. Responses ranged from 1=never to 4=always.

#### Physician’s cultural beliefs regarding cancer; perceived patient’s social and access barriers to screening

*Physicians’ cultural beliefs regarding cancer*: Was measured by the cultural beliefs regarding cancer subscale of the ACSB which was described above. The internal consistency of this subscale was .85 (Cronbach’s alpha).

*Perceived patients’ social barriers to screening*: Was measured by the social barriers to screening subscale of the ACSB, adjusted version for physicians. Physicians were asked to rate the extent to which each barrier to screening is true for their women patients. The internal consistency of this subscale was .81 (Cronbach’s alpha).

*Perceived patients’ access barriers to screening*: Was measured by the access barriers to screening subscale of the ACSB, adjusted version for physicians. Physicians were asked to rate the extent to which each barrier to screening is true for their women patients. Internal consistency (Cronbach’s alpha) was .84.

*The physician’s perception of patients’ barriers to mammography due to fear of pain* was assessed by one item probing the degree fear of the pain consists a barrier to undergoing mammography.

*The physician’s perceptions about patients’ barriers to mammography due to fear of radiation* were assessed by one item probing the degree fear of the radiation consists a barrier to undergoing mammography.

*The fatalistic beliefs scale* (Azaiza *et al*., 2010; 2011) was composed of four items: two items that related to fatalistic beliefs about breast cancer (e.g., breast cancer is a death sentence, despite the treatment); two items related to avoidance of cancer (i.e., it is better not to do the screening; ‘what you don’t know can’t hurt you’). Participants were asked to rate each question on a scale from 1=not at all to 5=very much. Internal consistency (Cronbach’s alpha) was .76.

### Data analysis

Descriptive statistics of the socio-demographic and study variables were conducted followed by *t*- and chi-square tests for the comparison in background and study variables between the two groups. Binary logistic regression analyses were conducted to identify the contribution of the cancer-related perceptions on physicians’ recommending and women adhering to mammography. Physicians’ recommendation variable was dichotomized into 1 = recommend to all or most women in the relevant age group 0 = none or some of the women. Adherence was defined as 1 = being on schedule and 2 = not being on schedule (see Measures section). Two models of logistic regression were tested, a model adjusted for age and religion and an unadjusted model that includes the study variables only. A *P* < 0.05 was considered significant.

All analyses were run using SPSS 20.0. Missing data were less than 1%; therefore, data imputation was not performed. Sample size was calculated according to Cohen’s formula: The sample size required to receive a medium effect size at alpha < 0.01 for a multiple regression logistic regression with seven independent variables is *N* = 102.

## Results

Table 1 shows the background details of the participants. The majority of the physicians were male (Arab women are underrepresented in the medical field in Israel (see Keshet *et al*., 2015), with a mean age of about 44 years. The physicians were predominantly Muslims; the rest were Christians (the ratio was proportional to the distribution of the Arab population in Israel (Central Bureau of Statistics, 2015), married, and mildly, or moderately religious. All of the physicians were employed in the community health services. About 70% specialized in family medicine; the remainder specialized in gynecology and internal medicine. The mean years of seniority were about 15, ranging from 1 to 42 years. The mean age of the women sample was 58, about 77% were married, similar to the physicians they were predominantly Muslims. Significant differences between the physician’s and women’s samples for all background variables. The women’s mean age was higher than that of the physicians. Similarly, women were more religious than the physicians. However, more physicians than women were married or had a partner, and more physicians than women were Muslims.

**Table 1.** Background variables: physicians’ sample and women’s sample

Variable	Physicians (*N* = 143)	Women (*N* = 290)	Difference
**Age, Years**, M (SD)	44.06 (11.36)	57.94 (6.11)	*t* (431) = 15.65**
Range	26–68	50–70	
**Family status**, N (%)			*χ* ^2^(1) = 5.97*
Married or intimate relations	121 (84.6)	225 (77.60)	
Single	22 (15.4)	65 (22.40)	
**Religion**, N (%)			*χ* ^2^(1) = 3.35*
Muslim	129 (90.2)	243 (83.79)	
Christian	14 (9.9%)	47 (16.21)	
**Religiosity**, M (SD)	2.14 (0.84)	2.67 (0.74)	*t* (432) = 4.98**
Range	1–4	1–4	

*P < .05; **P < .001

### Comparison between physicians’ reports of recommending mammography and laywomen’s reports that they were advised to undergo mammography

The majority (77.62 %; *N* = 111) of the physicians reported that they recommend annual or bi-annual mammography screening to all their women patients; 12.59% (*N* = 18) recommended screening to most of their patients. In addition, 75.52% (*N* = 108) said that they always discuss the advantages of regular mammography with non-adherent patients, 16.78% (*N* = 24) do so most of the time, and 7.69 (*N* = 11) do so seldom or never. In contrast, only 72.41% (*N* = 210) of the women participants recalled their family physician, gynecologist, or other specialized physician ever discussing or recommending undergoing a mammography test. Nevertheless, the vast majority (92.00%, *N* = 266) of the women participants had, at some time in the past, undergone a mammography test, 60.34% (*N* = 175) had performed a mammography during the previous two years and reported undergoing mammography every 1–2 years, in accordance with the guidelines (this figure includes women aged 50–52 years who said they had performed the test once, in compliance with the guidelines). In addition, the vast majority (93.70%, *n* = 134) of the physicians perceived mammography as effective or very effective, while 6.29% (*N* = 9) ascribed medium or low importance to conducting regular mammography. Similarly, 92.75% (*N* = 269) of the women perceived mammography as an effective or a very effective test.

### A comparison of beliefs and barriers to mammography screening between physicians and laywomen

An independent sample *t* test was performed to examine the differences in cultural and fatalistic beliefs, as well as in barriers to mammography screening (Table 2). Women held more cultural beliefs than physicians. Women also held more fatalistic beliefs than physicians. Physicians attributed more access barriers to screening as well as fear of radiation to women patients, compared with the women’s community sample. In contrast, women reported greater social barriers to breast cancer screening than were attributed to women patients by physicians. No significant differences were found in the perception of barriers to screening due to fear of pain between the two samples.

**Table 2.** Physicians and women cancer-related perceptions

	Physicians (*N* = 143)		Women (N = 290)		
Variable	M	SD	M	SD	*t*-test (df = 431)
Cultural beliefs	1.53	0.72	2.52	0.71	13.65*
Social barriers	1.87	.87	2.29	.89	5.58*
Access barriers	2.29	.81	1.89	1.11	3.84*
Fatalistic beliefs	1.46	.92	4.56	.81	34.04*
Fear of pain	2.46	1.02	2.35	1.45	0.81
Fear of radiation	2.71	1.07	1.65	1.5	9.49*

*P < .001

### The role of beliefs and barriers to mammography screening in physicians’ decision to recommend undergoing mammography and women adhering to mammography

A logistic regression was conducted in order to test the effect of physicians’ cancer-related perceptions on recommending mammography screening and the effect of cancer-related perceptions on women’s adherence to screening (see Table 3). First, an adjusted model was examined, including age and religion and the study variables: for the recommending mammography by physicians were entered the variables of their cultural beliefs and their perception of women’s social; barriers, access barriers, women’s fatalistic belief, and the barriers of fear of pain and radiation. For the women’s adherence with mammography guidelines, the independent variables were their own cultural perceptions, perceived social barriers, access barriers, fear of pain and radiation, and fatalistic beliefs. Physicians who had higher fatalistic beliefs and those who perceived that accessing barriers impede women’s adherence had a lower tendency to recommend mammography screening. Nevertheless, among physicians, higher perceived fear of pain as their patient’s barriers to attending mammography was related to a higher chance of recommending screening. In contrast, the only variable that had a significant effect on undergoing screening in the women’s group was fatalistic beliefs, while higher fatalistic beliefs were related to a lower chance of undergoing screening.

**Table 3.** Logistic regression of the effect of cancer-related perceptions on physicians’ recommending and women adhering to mammography

	Physicians (*N* = 143)				Women (*N* = 290)			
	Unadjusted OR		Adjusted OR		Unadjusted OR		Adjusted OR	
Variable	OR	95% CI	OR	95% CI	OR	95% CI	OR	95% CI
Cultural beliefs	0.82	0.43–12.56	0.99	0.48–2.00	1.12	0.66–1.90	0.96	0.53–1.71
Social barriers	0.64	0.34–1.18	0.94	0.47–1.87	0.55	0.34–0.84	0.97	0.63–1.47
Access barriers	0.19	0.08–1.16	0.28**	0.13–0.64	1.00	0.72–1.41	0.93	0.70–1.42
Fatalistic beliefs	0.56*	0.35–0.90	0.54*	0.32–0.89	0.81*	0.64–0.93	0.77*	0.60–0.92
Fear of pain	2.12*	1.16–3.87	2.33*	1.19–4.54	0.13	0.86–1.48	1.16	0.89–1.52
Fear of radiation	1.21	1.16–3.87	1.20	0.73–2.00	1.05	0.74–1.51	1.01	0.70–1.22

*P < .05; ** *P* < .01

## Discussion

This study examined the role of EMs of illness (cancer-related perceptions) in physicians’ and laywomen’s behaviors (decision to recommend undergoing regular mammography, adhering to mammography). As in many previous studies (e.g., Kessels, 2003; van der Meulen *et al*., 2008; Pianosi *et al*., 2016), our findings show gaps between physician-women’s reports concerning medical information. We found that the percent of women who recalled being advised by a physician to undergo a mammography was lower than the number of physicians who reported conveying such advice.

Furthermore, our findings might also suggest that women assign less significance to the mammography test than physicians. These findings are not surprising, but they are troubling, given the impact of the early detection of breast cancer on mortality (Gøtzsche and Jørgensen, 2013). Nevertheless, the majority of women reported undergoing a mammography test. This may be attributed to vigorous national efforts to increase adherence to screening through a population-based invitational breast cancer screening program and to educate women via the mass media. Although the physicians have an imperative role in reminding and encouraging to perform mammography and discussing barriers with patients, these findings may imply that, apart from physicians’ advice, other factors play a role in engaging in breast cancer screening behavior.

We also found evidence that physicians and laywomen hold different perceptions of cancer. This aligns with previous studies which show that physicians and patients/laypersons differ in their EMs or perceptions of illness, even if they share the same culture and language (e.g., Aido and Harpham, 2001; Weller *et al*., 2012). In other words, and in accord with other research on EM and illness perception among laypersons (Kleinman *et al*., 2006), in general, and cancer perception among Arabs and Arab women, in particular (Azaiza and Cohen, 2006; 2008; Cohen and Azaiza, 2005; 2008; 2010; Baron-Epel, 2010; Goldblatt *et al*., 2013; Cohen, 2014), the women’s perception of illness was shaped by cultural and religious beliefs of cancer (fatalistic beliefs and cultural beliefs).

Women’s perceptions were somewhat reserved concerning early detection outcomes; they tended to view cancer as a death verdict (Azaiza *et al*., 2010), a punishment inflicted by God, the price for wrongdoing, and/or a trial; and often found comfort in religious texts (Cohen and Azaiza, 2008). We found that laywomen also reported greater social barriers (fear of being pitied, of detachment and resentment from one’s husband, of being disrespected by one’s family, of losing one’s place of work and friends, and of neglecting one’s family) to breast cancer screening than were attributed to women patients by physicians. These barriers attest to the level of cancer-related stigma associated with cancer in the women’s communities, to their fear of being marked as socially inferior, and therefore excluded from mainstream activities and roles, especially the role of the Arab woman, who is first and foremost a spouse and mother (Azaiza and Cohen, 2008; Cohen, 2014).

The results show that physicians mistakenly attributed both access barriers and fear of radiation to their patients. The findings imply that physicians fail to address patients’ and laypeople’s perspectives of cancer in the medical encounter. While physicians put an emphasis on common or typical barriers to health among the general population such as fear (as is the case, e.g., in relation to dental care, see, e.g., Gordon *et al*., 1998) and accessibility (which is more prevalent among minority populations; see, e.g., Steinberg *et al*., 2006), the women reported cultural-related barriers. This might imply that if and when addressing barriers to screening, physicians tend to address the wrong set of barriers.

Our findings concerning the associations between perceptions and reported behavior are of particular importance, especially since they address the role of cultural perceptions among physicians. Fatalistic beliefs played a central role in undergoing mammography among the women. As was mentioned above, such beliefs are part and parcel of the women’s cultural and religious milieu, but it also implies that misunderstanding concerning the nature of cancer (a chronic disease) and novel treatments also play a role. Importantly, we found that physicians’ fatalistic beliefs (and the attribution of accessibility barriers) also affect their behavior. These findings are especially troubling and are in accord with other studies that illustrate how medical decisions may be prone to bias (Croskerry, 2013).

The study has several limitations, first and foremost is its design – a cross-sectional survey based on two individual samples rather than dyads or clusters of physician–patient/s. Future studies should address this limitation as well as replicate our study in other cultural groups. Future qualitative studies should examine the gaps in illness perceptions among physicians and patients and their implications. Second, the low physicians’ participation rate hampers the representability of the sample. Future studies should attempt to engage a higher response rate among physician samples. Third, the fact that mammography screening is readily offered to Israeli women may positively influence our findings. It is important to note that in less-developed countries where the health system lacks resources, there is an ongoing debate whether to implement mammography programs (Li and Shao, 2015). The WHO and others have therefore argued for adopting other screening methods (e.g., clinical examination Li and Shao, 2015; Black and Richmond, 2019). While this study as well as most studies (e.g., Cohen and Azaiza, 2008; Baron-Epel, 2010; Azaiza *et al*., 2011; Keinan-Boker *et al*., 2013; Cohen, 2014; Lavy *et al*., 2016) regarding health behaviors of Arab women in Israel and in the Palestinian Authority had not differentiated between subgroups of religions (e.g., Muslims and Christians) due to cultural and lifestyle similarities, it is suggested that further studies will assess patients–physicians’ perceptions and communication patterns in relation to health behaviors, by religion subgroups. Nevertheless, in spite of the limitations, our study is the first to compare physicians’ and layperson’s perceptions of cancer, in general, and among Arab-Israelis, in particular.

Our findings hold implications for practice and policy. The findings concerning the physician–patient–communication-related factors we studied suggest that there is room for improvement. Although today’s curriculum for medical students addresses the role of social, cultural, and behavioral factors in health (Dao, 2017), our findings suggest that further attention to this subject, in general, and to the role of cultural perceptions, in particular, are warranted. In addition to understanding the patient’s perceptions of illness, physicians should also reflect on the social, cultural, and psychological factors that shape his/her decision.

## Appendix 1:

**Table:** the Arab culture-specific barriers (ACSB)

To what extent the following is a difficulty for you in undergoing mammography?
**Environmental barriers**
Language and communication. Distance and accessibility of the clinic 16. Cost of the examinations
**Exposure barriers**
A male physician examining my breast embarrasses me A female physician examining my breast embarrasses me Body exposure is forbidden by religion. I fear being seen at the clinic
**Social barriers**
Fear pity by others Fear disrespect by my family Fear losing my job Fear my husband's detachment, resentment Fear neglecting my family Fear losing my friends
**Cultural beliefs concerning cancer**
Cancer is a way of punishment by God Cancer is a way of atonement for bad deeds by God Cancer is a trial by God Reading religious writings assists healing

## References

[ref1] Aidoo M and Harpham T (2001) The explanatory models of mental health amongst low-income women and health care practitioners in Lusaka, Zambia. Health Policy and Planning 16, 206–213. 11358923 10.1093/heapol/16.2.206

[ref2] Ashton CM , Haidet P , Paterniti DA , Collins TC , Gordon HS , O’Mailley K , Petersen LA , Sharf BF , Suarez-Almazor ME , Wray NP and Street Jr RL (2003) Racial and ethnic disparities in the use of health services. Journal of General Internal Medicine 18, 146–152. 12542590 10.1046/j.1525-1497.2003.20532.x PMC1494820

[ref3] Azaiza F and Cohen M (2006) Health beliefs and rates of breast cancer screening among Arab women. Journal of Women’s Health 15, 520–530. 10.1089/jwh.2006.15.520 16796479

[ref4] Azaiza F and Cohen M (2008) Between traditional and modern perceptions of breast and cervical cancer screenings: a qualitative study of Arab women in Israel. Psycho-Oncology 17, 34–41. 17352007 10.1002/pon.1180

[ref5] Azaiza F , Cohen M , Awad M and Daoud F (2010) Factors associated with low-screening for breast cancer in the Palestinian Authority. Cancer 116, 4646–4655. 20589933 10.1002/cncr.25378

[ref6] Azaiza F , Cohen M , Daoud F and Awad M (2011) Traditional-Westernizing continuum of change in screening behaviors: comparison between Arab women in Israel and the West Bank. Breast Cancer Research and Treatment 128, 219–227. 21191648 10.1007/s10549-010-1321-1

[ref7] Baer RD , Weller SC , García JGDA and Rocha ALS (2004) A comparison of community and physician explanatory models of AIDS in Mexico and the United States. Medical Anthropology Quarterly 18, 3–22. 15098425 10.1525/maq.2004.18.1.3

[ref8] Baer RD , Weller SC , García JGDA and Rocha ALS (2008) Cross-cultural perspectives on physician and lay models of the common cold. Medical Anthropology Quarterly 22, 148–166. 18717364 10.1111/j.1548-1387.2008.00012.x

[ref9] Baron-Epel O (2010) Attitudes and beliefs associated with mammography in a multiethnic population in Israel. Health Education & Behavior 37, 227–242. 19690289 10.1177/1090198109339460

[ref10] Black E and Richmond R (2019) Improving early detection of breast cancer in sub-Saharan Africa: why mammography may not be the way forward. Globalization and Health 15, 3. 30621753 10.1186/s12992-018-0446-6 PMC6325810

[ref11] Central Bureau of Statistics (2015) *The Arab population 2008.* Retrieved 30 January 2019 from http://www.cbs.gov.il/www/statistical/arab_pop08.pdf

[ref12] Champion VL (1999) Revised susceptibility, benefits, and barriers scale for mammography screening. Research in Nursing & Health 22, 341–348. 10435551 10.1002/(sici)1098-240x(199908)22:4<341::aid-nur8>3.0.co;2-p

[ref13] Croskerry P (2013) From mindless to mindful practice – cognitive bias and clinical decision making. New England Journal of Medicine 368, 2445–2448. 23802513 10.1056/NEJMp1303712

[ref14] Cohen M (2013) Cancer fatalism: attitudes toward screening and care In Carr BI and Steel J , editors, Psychological aspects of cancer. New York: Springer, 83–99.

[ref15] Cohen M (2014) An integrated view of cultural perceptions of cancer among Arab people in Israel. Health Psychology Review 8, 490–508. 25211212 10.1080/17437199.2013.816205

[ref16] Cohen M and Azaiza F (2005) Early breast cancer detection practices, health beliefs, and cancer worries in Jewish and Arab women. Preventive Medicine 41, 852–858. 16120457 10.1016/j.ypmed.2005.07.001

[ref17] Cohen M and Azaiza F (2008) Developing and testing an instrument for identifying culture-specific barriers to breast cancer screening in Israeli Arab women. Acta Oncologica 47, 1570–1577. 18607884 10.1080/02841860802078069

[ref18] Cohen M and Azaiza F (2010) Increasing breast examinations among Arab women using a tailored culture-based intervention. Behavioral Medicine 36, 92–99. 20801757 10.1080/08964280903521313

[ref19] Dao DK , Goss AL , Hoekzema AS , Kelly LA , Logan AA , Mehta SD , Sandesara UN , Munikwa MR and DeLisser HM (2017) Integrating theory, content, and method to foster critical consciousness in medical students: a comprehensive model for cultural competence training. Academic Medicine 92, 335–344. 27680318 10.1097/ACM.0000000000001390

[ref20] Goldblatt H , Cohen M , Azaiza F and Manassa R (2013) Being within or being between? The cultural context of Arab women’s experience of coping with breast cancer in Israel. Psycho-Oncology 22, 869–875. 22473753 10.1002/pon.3078

[ref21] Gordon SM , Dionne RA and Snyder J (1998) Dental fear and anxiety as a barrier to accessing oral health care among patients with special health care needs. Special Care in Dentistry 18, 88–92. 9680917 10.1111/j.1754-4505.1998.tb00910.x

[ref22] FGøtzsche PC and Jørgensen KJ (2013) Screening for breast cancer with mammography. Cochrane Database of Systematic Reviews 6, CD001877. 10.1002/14651858.CD001877.pub5 PMC6464778 23737396

[ref23] Ferlay J , Shin HR , Bray F , Forman D , Mathers C and Parkin DM (2010) Estimates of worldwide burden of cancer in 2008: GLOBOCAN 2008. International Journal of Cancer 127, 2893–2917. 21351269 10.1002/ijc.25516

[ref24] Haidet P , O’Malley KJ , Sharf BF , Gladney AP , Greisinger AJ and Street RL Jr (2008) Characterizing explanatory models of illness in healthcare: development and validation of the CONNECT instrument. Patient Education and Counseling 73, 232–239. 18760889 10.1016/j.pec.2008.07.007

[ref25] Israel Cancer Association (2015) *Early Detection and Prevention of Breast Cancer* 2015. Retrieved 30 January 2019 from http://www.cancer.org.il/template/default.aspx?PageId=5963

[ref26] Keinan-Boker L , Baron-Epel O , Fishler Y , Liphshitz I , Barchana M , Dichtiar R and Goodman M (2013) Breast cancer trends in Israeli Jewish and Arab women, 1996–2007. European Journal of Cancer Prevention 22, 112–120. 23361380 10.1097/CEJ.0b013e3283581d3c

[ref27] Keshet Y , Popper-Giveon A and Liberman I (2015) Intersectionality and underrepresentation among health care workforce: the case of Arab physicians in Israel. Israel Journal of Health Policy Research 4, 18. 25878770 10.1186/s13584-015-0004-0 PMC4397687

[ref28] Kessels RP (2003) Patients’ memory for medical information. The Journal of the Royal Society of Medicine 96, 219–222. 12724430 10.1258/jrsm.96.5.219 PMC539473

[ref29] Kleinman A (1980) Patients and healers in the context of culture. Berkeley, CA: University of California Press.

[ref30] Kleinman A , Eisenberg L and Good B (2006) Culture, illness, and care: clinical lessons from anthropologic and cross-cultural research. Focus 4, 140–149 10.7326/0003-4819-88-2-251 626456

[ref31] Lavy R , Hershkovitz Y , Keinan-Boker L and Halevy A (2016) Incidence and trends of gastrointestinal malignancies in Jewish and Arab populations in Israel over 32 Years. Israeli Medical Association Journal 18, 466–469. 28471577

[ref32] Li J and Shao Z (2015) Mammography screening in less developed countries. SpringerPlus 4, 615. 26543750 10.1186/s40064-015-1394-8 PMC4627993

[ref33] Lupton D (2012) Medicine as culture: illness, disease and the body. London: Sage.

[ref34] Nowicka-Sauer K , Pietrzykowska M , Banaszkiewicz D , Hajduk A , Czuszyńska Z and Smoleńska Ż (2016) How do patients and doctors-to-be perceive systemic lupus erythematosus? Rheumatology International 36, 725–729. 26873361 10.1007/s00296-016-3431-5

[ref35] Oeffinger KC , Fontham ET , Etzioni R , Herzig A , Michaelson JS , Shih YCT , Walter LC , Church TR , Flowers CR , LaMonte SJ , Wolf AMD , DeSantis C , Lortet-Tieulent J , Andrews K , Manassaram-Baptiste D , Saslow D , Smith RA , Brawley OW and Wender R (2015) Breast cancer screening for women at average risk: 2015 guideline update from the American Cancer Society. JAMA 314, 1599–1614. 26501536 10.1001/jama.2015.12783 PMC4831582

[ref36] Pianosi K , Gorodzinsky AY , Chorney JM , Cosrten G , Johnson LB and Hong P (2016) Informed consent in pediatric otolaryngology: what risks and benefits do parents recall? Otolaryngology – Head and Neck Surgery 155, 332–339. 27048666 10.1177/0194599816641910

[ref37] Rich M , Patashnick J and Chalfen R (2002) Visual illness narratives of asthma: explanatory models and health-related behavior. American Journal of Health Behavior 26, 442–453. 12437019 10.5993/ajhb.26.6.5

[ref38] Schouten BC and Meeuwesen L (2006) Cultural differences in medical communication: a review of the literature. Patient Education and Counseling 64, 21–34. 16427760 10.1016/j.pec.2005.11.014

[ref39] Steinberg AG , Barnett S , Meador HE , Wiggins EA and Zazove P (2006) Health care system accessibility. Journal of General Internal Medicine 21, 260–266. 16499543 10.1111/j.1525-1497.2006.00340.x PMC1828091

[ref40] Tirodkar MA , Baker DW , Makoul GT , Khurana N , Paracha MW and Kandula NR (2011) Explanatory models of health and disease among South Asian immigrants in Chicago. Journal of Immigrant and Minority Health 13, 385–394 20131000 10.1007/s10903-009-9304-1 PMC2905487

[ref41] van der Meulen N , Jansen J , van Dulmen S , Bensing J and van Weert J (2008) Interventions to improve recall of medical information in cancer patients: a systematic review of the literature. Psycho-Oncology 17, 857–868. 18050149 10.1002/pon.1290

[ref42] Weller SC , Baer RD , Garcia JGDA and Rocha ALS (2012) Explanatory models of diabetes in the US and Mexico: the patient–provider gap and cultural competence. Social Science & Medicine 75, 1088–1096. 22703883 10.1016/j.socscimed.2012.05.003

[ref43] Zoabi K and Savaya R (2017) Cultural identity and intervention strategies of Arab minority social workers in Israel. The British Journal of Social Work 47, 392–408.

